# Comparing the effects of breastfeeding in the laid-back and cradle position upon the experiences of primiparous women: a parallel randomized clinical trial

**DOI:** 10.1186/s13063-023-07143-0

**Published:** 2023-02-13

**Authors:** Asefe Bashiri, Leila Amiri-Farahani, Hamid Salehiniya, Sally Pezaro

**Affiliations:** 1 Department of Reproductive Health and Midwifery, School of Nursing and Midwifery, Iran University of Medical Sciences, Tehran, Iran; 2 Nursing and Midwifery Care Research Center, Department of Reproductive Health and Midwifery, School of Nursing and Midwifery, Iran University of Medical Sciences, Tehran, Iran; 3 Social Determinants of Health Research Center, Birjand University of Medical Sciences, Birjand, Iran; 4 The University of Notre Dame, Australia and Assistant Professor, The Centre for Healthcare Research, Coventry University, Coventry, UK

**Keywords:** Laid-back position, Cradle position, Breastfeeding experience, Breastfeeding positions

## Abstract

**Background and aim:**

Appropriate positioning is crucial to successful breastfeeding and its continuation. Positioning can create, prevent, or correct breastfeeding problems. This study aimed to determine and compare the effects of both the laid-back and cradle positions upon the breastfeeding experiences of primiparous (cisgender) women.

**Methods:**

A parallel randomized clinical trial was conducted with a sample of primiparous women (*n* = 168) with a gestational age of between 31 and 34 weeks, referring to the perinatal clinic of Shahid Gharazi Hospital in Malayer (Hamadan Province, Iran). Participants were recruited via convenience sampling and allocated to one of two groups: intervention (laid-back position) (*n* = 85) and control (cradle position) (*n* = 83) using the random block method. Breastfeeding education was given to both groups during two sessions (weeks 31–34 and 35-–37 of pregnancy) by “baby-friendly” accredited hospitals in Iran. The intervention group was instructed on how to breastfeed using the laid-back position. The control group was instructed on how to breastfeed using the cradle position. In the immediate postnatal period, breastfeeding positions were assessed in both groups. Breastfeeding experiences were measured once at the time of participants returning home and again at both 1 week and 2 weeks following birth, using the breastfeeding experience scale. Data analysis was undertaken using the SPSS software version 21. A value of *P* < 0.05 was considered significant.

**Results:**

No statistically significant difference was observed between the two groups in terms of concerns relating to participants nor their breasts, milk insufficiency, neonate, process, or breastfeeding experience at any time point measured. The means (SDs) of breastfeeding experience at the time of discharge, 1 week, and 2 weeks after childbirth in intervention group were 26.07 (4.533), 26.85 (3.812), and 26.65 (4.632) respectively. The means (SDs) of breastfeeding experience at the time of discharge, 1 week, and 2 weeks after childbirth in control group were 25.42 (3.315), 26.68 (3.872), and 25.41 (4.05) respectively.

**Conclusion:**

There is no difference in breastfeeding experiences whether the laid-back or cradle position is used. Thus, broader education on breastfeeding and the provision of comprehensive support may be more effective in optimizing one’s experience of breastfeeding.

**Trial registration:**

Registration date: 2021 July 21, Code: IRCT20180427039436N10, https://irct.ir/user/trial/57054/view

**Supplementary Information:**

The online version contains supplementary material available at 10.1186/s13063-023-07143-0.

## Introduction

The process of breastfeeding can have a unique emotional and biological effect on the health of both the breast feeder and the infant [1]. The World Health Organization has highlighted the importance of exclusive breastfeeding (EBF) in the first 6 months of life and its continuation until 2 years of age [2]. Breastfeeding has significant benefits to the health of both parents and their infants [3–5]. For example, breastfeeding reduces babies’ possibility of developing chronic diseases such as type II diabetes in adulthood [6] and cancer in childhood [7]. For the breast feeder, breastfeeding reduces their risk of high blood pressure [8], type II diabetes [9], and breast and ovarian cancer [10].

Despite recommendations from the WHO, only 40% of children under 6 months old are exclusively breastfed [11]. Yet, a systematic review and meta-analysis conducted in Iran demonstrated that the overall prevalence of exclusive feeding was 53% in 2017 [12].

The development of breastfeeding problems can lead to breastfeeding children less [13]. Such problems are largely affected by the breastfeeding method used. One’s method of breastfeeding can be described as the combination of both position and attachment; position refers to a technique in which the infant is kept in relation to the body, and attachment is related to the infant putting the areola and breast tissue in its mouth effectively [14].

Improper positioning can lead to improper attachment of the infant during breastfeeding [15], and improper attachment increases the possibility of sores and cracked nipples [16]. Among other problems that occur due to incorrect position or attachment are feelings of tiredness and insufficient supply of breast milk, which are common problems in the postpartum period [17, 18]. Pain and injury to the nipple and fissure may also occur due to inappropriate breastfeeding techniques and inappropriate positioning [19]. Such findings point to the need for further investigation as to which types of positioning may be most effective and how breastfeeding education may be most useful in this context.

There are many common positions in which to breastfeed. Milinco et al. compared usual breastfeeding positions with the laid-back position, also known as biological nurturing. Results demonstrated that breastfeeding problems in the laid-back position group decreased compared to those engaging their usual breastfeeding positions but had no effect on the frequency of EBF and satisfaction [20].

In another study, the frequency of nipple pain and nipple trauma in a group employing the laid-back breastfeeding position was lower than in those employing their usual breastfeeding positions, yet no statistically significant difference was observed between the two groups in relation to the comfort and “correctness” of the positioning [21]. Similarly, in a different study, the frequency of nipple trauma in the laid-back breastfeeding position group was lower in than the usual breastfeeding position group, and no statistically significant difference was observed between the two groups in terms of comfort and correct attachment [22]. Yet, in the study of Puapornpong et al., those using the laid-back and side-lying breastfeeding positions had no significant differences in latching and EBF rates. However, there was greater satisfaction with comfort, ease of positioning, and breastfeeding for long periods in the side-lying group compared to the laid-back group [23].

Evidence in relation to positioning during breastfeeding are conflicting and lacking. For example, in one meta-analysis, results remain unclear and contradictory, particularly in relation to comfort in positioning [24]. This uncertainty in evidence is further highlighted in the systematic review conducted by Guille et al., in which it has been suggested that an interventional study design is now required [25]. Considering the above and the lack of evidence in relation to Iran in particular, the present study aimed to compare the effect of two breastfeeding positions (laid-back and cradle) on breastfeeding experience and its subscales among primiparous (cisgender) women in Iran. The objectives of this study were to compare maternal concerns, breast concerns, milk insufficiency concerns, neonatal concerns, process concerns, and breastfeeding experience in primiparous women breastfeeding in both the laid-back and cradle positions at the time of discharge, 1 and 2 weeks after childbirth.

## Methods

### Design, setting, randomization, blinding

This study is a parallel randomized clinical trial including an intervention and control group. Reporting of this study is in accordance with the Consolidation Standards of Reporting Trials (CONSORT) statement (Supplementary file 1) [26]. This study has been funded by the Iran University of Medical Sciences. The study’s protocol was registered in the Clinical Trial Registration Center on July 21, 2021, with the code: IRCT20180427039436N10. The study population consisted of women between the 31st and 34th week of pregnancy attending the perinatal clinic of Shahid Gharazi Hospital in Malayer (Hamadan Province, Iran).

The researchers first identified eligible individuals using convenience sampling and provided them with the necessary explanations on the study process. Those who agreed to take part were then invited to give their informed consent and enrolled. The recruitment period lasted 6 months. The intervention period began in August 2021. Follow-up data collection ended in January 2022.

Eligible participants were assigned to one of two groups (intervention and control) using the block randomization method at a ratio of 1:1 (available at www.randomization.com). To determine the sequence of participants’ allocation based on the block balanced randomization method, the total sample size must first be ascertained along with the number of groups, and the number of group repetitions in each block (considered equal). In the current study, the size of each block was twice as big as the number of groups (4 groups in each block) [27]. An epidemiologist, who was not part of the study, constructed the randomization list. For allocation concealment, the research team had no access to this list.

Blinding was not possible due to the nature of the interventions. Yet, the data collection tool was used by a research assistant who had no knowledge of the study groups and their allocation. Furthermore, statistical analysis was performed by a statistician who also had no knowledge of the content provided for each of the study groups and their allocation.

### Participants

Participants met the inclusion criteria where they had experienced a singleton pregnancy, a desire to exclusively breastfeed, given birth in Shahid Gharazi Hospital, and a gestational age of 37–42 weeks at the time of birth. Moreover, to be included, the Apgar score of the infant in the first and fifth minutes was required to be greater than or equal to 7, and its weight was required to be between 2500 and 4000 g. Participants met the exclusion criteria where congenital abnormalities were incompatible with breastfeeding (e.g., abnormalities in the mouth, palate, tongue, jaw, and face) and breastfeeding was contraindicated (e.g. non-prescription drug use during breastfeeding or mastectomy). Participants were withdrawn from the study where they required hospitalization in an intensive care unit after birth, used other breastfeeding positions, and/or did not complete questionnaires 1 week and/or 2 weeks after childbirth.

### Sample size

Sample size was estimated based on the work of Puapornpong et al., considering the mean of outcome in the two groups to be equal to 3.8 and 4, respectively, and the standard deviation to be 0.6 and 0.1 [23] with a power of 80% and a confidence interval of 95%, and the sample size was calculated to be 76 participants in each group using the following formula (means comparison):

$$\begin{array}{cc}n=\frac{{\left({Z}_{1-\alpha /2}+{Z}_{1-\beta }\right)}^{2}\left({S}_{1}^{2}+{S}_{2}^{2}\right)}{{\left({\mu }_{1}-{\mu }_{2}\right)}^{2}}& n=\frac{{\left(1.96+0.85\right)}^{2}\left(0.36+0.01\right)}{0.04}\end{array}=76$$

However, 84 participants were assigned to each group in anticipation of a 10% drop in sample size through attrition.

### Assessment of trial variables

An individual characteristics questionnaire was used to collect demographic information. In order to answer the main outcome variable of the current study, the Breastfeeding Experience Scale (BES) was used [28, 29].

#### Individual characteristics questionnaire

The individual characteristics questionnaire consisted of 3 parts. The first part collected data in relation to the personal characteristics of the participant. The second part collected data in relation to pregnancy and childbirth. The third part collected data in relation to the infant. Individual characteristics and pregnancy information were completed upon recruitment to the study (31–34 weeks of pregnancy). Individual characteristics included questions about age, education level and occupation, education level and occupation of the spouse, economic status of the family, place of residence, and gestational age at the time of entering the study. Pregnancy information included the total number of previous pregnancies, number of previous abortions, and the gestational age in weeks at the time of study entry. Information related to childbirth included mode of birth and gestational age at the time of birth. Infant characteristics included assumed gender, weight, and Apgar scores of the first and fifth minutes of birth, which were collected by the researcher from medical records.

#### Main outcome variable

The outcome of the intervention related to breastfeeding experience. This was measured using the BES at three data collection points (discharge home, and 1 and 2 weeks after childbirth). The scales were completed by participants at home and returned to the research team.

BES is a questionnaire that consists of 30 items. The first 18 items measure presence or absence and severity of common breastfeeding difficulties in the early postpartum period. Scores range from “not at all” (1) to “unbearable” (5). The total score ranges from 18 to 90, with a higher score representing increased problem severity. The scale includes five subscales as follows: breast concerns, neonatal concerns, milk insufficiency concerns, maternal concerns, and process concerns. The last 12 items of the BES assess whether breastfeeding was continued, whether formula was added or substituted breast milk, how often formula was introduced, and what breastfeeding difficulties were related to parental weaning decisions in cases of early weaning [28, 29].

The validity of the English version and its reliability has been confirmed by the internal consistency method with Cronbach’s alpha of 0.77 [28]. The validity of the Persian version of the BES and the reliability of it has been confirmed in terms of internal consistency using Cronbach’s alpha coefficient of 0.83 [29]. In the current study, we used the first part of BES (18-item part).

### Intervention

The first and second parts of the breastfeeding education sessions were provided by the researcher when pregnant participants were between 31–34 and 35–37 weeks of pregnancy, respectively. The contents of breastfeeding education were designed according to a guidebook for monitoring “baby-friendly” hospitals in Iran [30, 31]. The information provided in educational sessions was the same for both groups. The educational program for both groups at 31–34 weeks gestation consisted of informing parents on the benefits of human milk, the importance of EBF, symptoms of starvation and fullness, breastfeeding on demand, breastfeeding from each breast, how to latch the infant on to the breast, and, finally, dispelling myths in relation to breastfeeding. The breastfeeding education delivered at 35–37 weeks of gestation included informing parents about breast pumping, common breast concerns and their prevention and treatment, ways to diagnose the adequacy of milk, support, ways to increase milk supply, along with issues related to bottle feeding, formula, pacifiers, diseases and breastfeeding, and drug use during breastfeeding [30, 31]. The only difference in education related to positioning, where one group were instructed on using the laid-back breastfeeding position in person, and the other were instructed on using the cradle breastfeeding position via a training video (healthy baby prepared by the Ministry of Health) [30, 31]. At the end of each session, all participant questions were answered. All participants also received educational pamphlets approved by the Education Committee at Shahid Gharazi Hospital. Also, the training was conducted by researcher (A.B).

After giving birth, participants’ breastfeeding practice commenced according to the taught breastfeeding positions. Breastfeeding support was offered as required and participants could contact the researcher for additional support if they experienced breastfeeding issues. The two groups were located in separate rooms. On the 3rd, 5th and 7th day of the postnatal period, participants were contacted by telephone to confirm the assigned breastfeeding position being used.

### Statistical analysis

Statistical analysis was performed using the SPSS version 21 software. Absolute and relative frequency and mean and standard deviation were used to analyze the descriptive data of the distribution. The normality of the data was checked with the Kolmogorov–Smirnov test. Independent *t*-test was used to compare quantitative variables between the two studied groups, and chi-square test and Fisher’s exact test were used to analyze qualitative variables. Repeated measures ANOVA were used for within-group comparisons. The effect sizes in between groups comparisons were reported based on Cohen’s *d*, and standardized mean difference was reported based on Cohen’s *d* effect size (null effect = 0, trivial effect = 0–0.19, small effect = 0.2 – 0.49, medium effect = 0.5–0.79, large effect = 0.8–1.19, very large effect = 1.2–2, and huge effect ≥ 2), [32]. A value of *P* < 0.05 was considered significant.

### Ethical considerations

The protocol of the present study was approved by the Ethics Committee of Iran University of Medical Sciences with the ethics code: IR.IUMS.REC.1400.297 and Iranian registry of clinical trials with code: IRCT201842739436N10. All participants were fully informed about the objectives and process of the study and informed written consent was obtained from them. The information obtained during the study process remained completely confidential. All patients were told that they could withdraw from the study at any time. No fees were paid for the study and all services were completely free.

## Results

### Samples’ characteristics

The primiparous pregnant women (*n* = 168) who participated in this study did so as follows: control group (*n* = 83) and intervention group (*n* = 85). During the study, there were 12 participants in the control group excluded from the study due to either hospitalization of either the participant or the infant, the weight of the infant being less than 2500 g or weight of the infant being more than 4000 g, having a gestational age of less than 37 weeks, and birthing in an alternate location. From the intervention group, 4 participants were excluded from the study due to their infant’s weight being over 4000 g and having a gestational age of less than 37 weeks at the time of birth and due to having birthed in an alternate location.

**Fig. 1:**
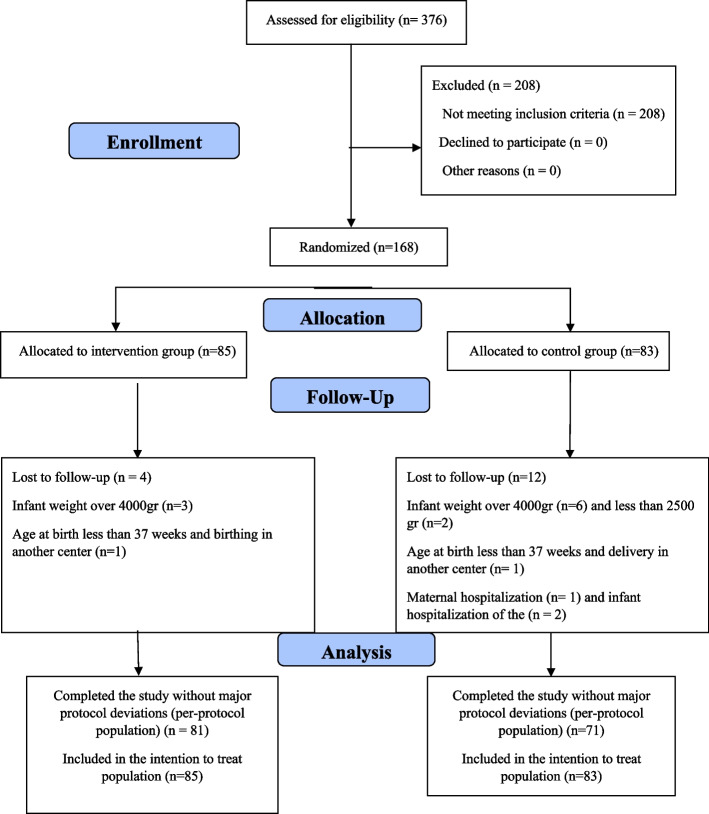
Enrolment of participants into two groups: intervention and control

Overall, the information of 152 primiparous women (71 participants in the control group and 81 participants in the intervention group) were analyzed (Fig. 1). Based on individual characteristics, there was no statistically significant difference identified between the two groups (Table 1).

**Table 1:** Participants’ characteristics and tests used to compare differences between the two groups

**Variables**	**Intervention group (*n* = 85)**	**Control group (*n* = 83)**	^a^*P* value
**Maternal age** (year), mean (SD^b^)	25.69 (5.46)	27.10 (6.41)	0.129
**Education status**, *n* (%)			0.81
Elementary	3 (3.53)	2 (2.41)	
Secondary	10 (11.76)	13 (15.67)	
High School	35 (41.18)	30 (36.14)	
University	37 (43.53)	38 (45.78)	
**Occupation status**, *n* (%)			1.000
Housewife	77 (90.59)	75 (90.36)	
Employed	8 (9.41)	8 (9.64)	
**Spouses’ education status**, *n* (%)			0.466
Elementary	3 (3.53)	1 (1.21)	
Secondary	7 (8.24)	8 (9.64)	
High school	31 (36.47)	38 (45.78)	
University	44 (51.76)	36 (43.37)	
**Spouses’ occupation status**, *n* (%)			0.108
Employed	22 (25.88)	13 (15.66)	
Self-employed	50 (58.83)	59 (71.09)	
Worker	10 (11.76)	11 (13.25)	
Unemployed	3 (3.53)	0 (00.00)	
**Economic status**, *n* (%)			0.679
Less than enough	10 (11.77)	7 (8.43)	
Enough	64 (75.29)	67 (80.72)	
More than enough	11 (12.94)	9 (10.85)	
**Place of residence**, *n* (%)			0.84
Urban	71 (83.52)	68 (81.92)	
Rural	14 (16.48)	15 (18.08)	
**Mode of birth**, *n* (%)			0.44
Vaginal	47 (55.30)	40 (48.19)	
Cesarean section	38 (44.70)	43 (5181)	
**Gestational age at beginning of study**, mean (SD)	32.11 (1.047)	32.06 (0.967)	0.77
**Gestational age at birth**, mean (SD)	38.85 (1.097)	39.13 (1.079)	0.091
**Number of abortions**, *n* (%)			0.61
0	66 (77.65)	59 (71.09)	
1	15 (17.65)	23 (27.71)	
2 and 3	4 (4.70)	1 (1.20)	
**Infant weight**, mean (SD)	3284.12 (387.929)	3337.35 (469.941)	0.424
**Assumed gender of infant**, *n* (%)			0.541
Girl	42 (49.41)	37 (44.58)	
Boy	43 (50.59)	46 (55.42)	
**Apgar first minute**, mean (SD)	8.95 (0.263)	8.88 (0.787)	0.416
**Apgar fifth minute**, mean (SD)	10.00 (0.000)	9.98 (0.220)	0.313

^a^*P* < 0.05 is significant

^b^Standard deviation

**Fig. 2:**
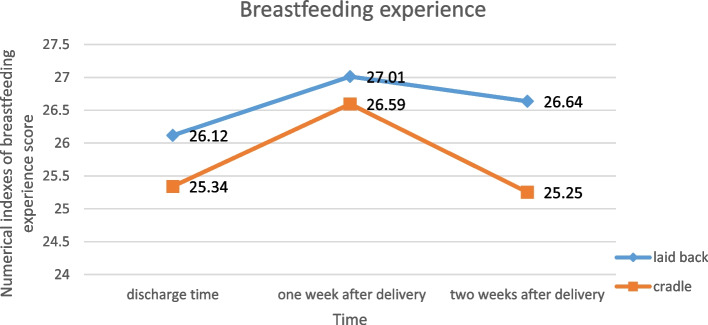
Comparison the mean of breast-feeding experience scores between the two groups at the times of hospital discharge and 1 and 2 weeks after childbirth

In between-group comparisons, the results of the independent sample *t*-test showed there are no statistically significant differences in terms of the mean scores of maternal concerns, breast concerns, milk insufficiency concerns, neonatal concerns, process concerns, and breastfeeding experience either at the time of discharge from hospital or at 1 and 2 weeks post birth. The results of within-group comparisons at the time of discharge, 1 week, and 2 weeks after childbirth in the control and intervention groups are given separately in Table 2, and Fig. 2, supplementary figures 1, 2, 3, 4 and 5.

**Table 2:** Effect of intervention on outcomes—per-protocol sample

**Variables**	**Intervention group (*n* = 81)**	**Control group (*n* = 71)**	**MD**^b^** (CI**^c^** 95%)**	**ES**^d^** (between)**	^e^***P*-value (between groups)**
	Mean (SD^a^)	Mean (SD^a^)			
**Maternal concerns**					
Time 1	6.38 (1.347)	6.00 (1.276)	0.383 (− 0.039 to 0.805)	0.289	0.075
Time 2	6.38 (1.538)	6.04 (1.367)	0.340 (− 0.129 to 0.810)	0.233	0.154
Time 3	6.73 (2.110)	6.41 (1.793)	0.320 (0.312 to 0.952)	0.163	0.319
^e^*P*-value (within groups)	0.171	0.1			
**Breast concerns**					
Time 1	4.38 (0.815)	4.38 (0.763)	0.002 (− 0.252 to 0.257)	0	0.985
Time 2	5.31 (1.158)	5.62 (1.438)	− 0.311 (− 0.727 to 0.105)	0.239	0.142
Time 3	4.84 (1.101)	4.66 (1.146)	0.178 (− 0.183 to 0.538)	0.16	0.332
*P*-value (within groups)	< 0.001	< 0.001			
*P*-value (two by two comparisons, times 1–2, 1–3, 2–3)	< 0.001,0.015,0.013	< 0.001, 0.266, < 0.001			
**Milk insufficiency concerns**					
Time 1	5.46 (2.515)	5.27 (2.164)	0.189 (− 0.568 to 0.947)	0.081	0.622
Time 2	3.83 (1.523)	4.21 (1.621)	− 0.384 (− 0.888 to 0.120)	0.242	0.134
Time 3	3.99 (1.714)	3.94 (1.539)	0.044 (− 0.481 to 0.569)	0.031	0.869
*P*-value (within groups)	< 0.001	< 0.001			
*P*-value (two by two comparisons, times 1–2, 1–3, 2–3)	< 0.001, < 0.001, 0.907	< 0.001, < 0.001, 0.174			
**Neonatal concerns**					
Time 1	5.37 (1.854)	5.21 (1.638)	0.159 (− 0.405 to 0.723)	0.091	0.578
Time 2	4.73 (1.285)	4.69 (1.077)	0.038 (− 0.345 to 0.421)	0.034	0.844
Time 3	4.60 (1.169)	4.30 (0.595)	0.309 (0.005 to 0.613)	0.317	0.046
*P*-value (within groups)	< 0.001	< 0.001			
*P*-value (two by two comparisons, times 1–2, 1–3, 2–3)	0.007, 0.001, 1.000	0.021, < 0.001, 0.002			
**Process concerns**					
Time 1	3.30 (1.112)	3.32 (1.039)	− 0.028 (− 0.374 to 0.319)	0.019	0.875
Time 2	5.22 (1.323)	4.75 (1.421)	0.476 (0.036 to 0.916)	0.343	0.034
Time 3	5.32 (1.499)	4.92 (1.592)	0.405 (− 0.090 to 0.901)	0.259	0.108
*P*-value (within groups)	< 0.001	< 0.001			
*P*-value (two by two comparisons, times 1–2, 1–3, 2–3)	< 0.001, < 0.001, 1.000	< 0.001, < 0.001, 1.000			
**Breastfeeding experience**					
Time 1	26.07 (4.533)	25.42 (3.315)	0.652 (− 0.637 to 1.940)	0.162	0.319
Time 2	26.85 (3.812)	26.68 (3.872)	0.176 (− 1.058 to 1.409)	0.044	0.779
Time 3	26.65 (4.632)	25.41 (4.05)	1.246 (− 0.158 to 2.649)	0.284	0.081
*P*-value (within groups)	0.248	0.016			

^a^Standard deviation

^b^Mean difference

^c^Confidence interval

^d^Effect size (ES) based on Cohen’s *d*

^e^*P*-value < 0.05 considerd significant

## Discussion

The aim of the present study was to compare the effect of two breastfeeding positions, laid-back and cradle positions on breastfeeding experience and its subscales among primiparous (cisgender) women in Iranian setting. The results showed no statistically significant difference between the two groups in terms of maternal concerns, breast concerns, milk insufficiency concerns, neonatal concerns, process concerns, and breastfeeding experience at the time of discharge and at both 1 and 2 weeks post childbirth.

In line with the results presented here, the results of Milinco et al.’s study using the Maternal Breastfeeding Evaluation Scale (MBFES) equally showed no statistically significant difference in satisfaction between participants’ breastfeeding in either the laid-back or the usual positions at the time of discharge from hospital and at 1 and 4 months post discharge [20]. Likewise, Wang et al.’s (2021) meta-analysis showed no statistically significant difference between the groups using laid-back breastfeeding positioning and traditional positioning in terms of comfort [24]. Thus, our results add to the body of evidence demonstrating that positioning has no significant effect in this area.

Nevertheless, the clinical trial conducted by Puapornpong et al. (2017) demonstrated that satisfaction scores were higher in the side-lying group than the laid-back group of participants who had given birth via cesarean section, and a statistically significant difference was observed between the two groups [23]. The outcome of maternal satisfaction in the Puapornpong et al.’s study is not consistent with the outcome of maternal concerns in the present study. The side-lying position is commonly used following cesarean birth as it prevents the pressure exerted by the weight of the infant on the abdomen [33]. Joshi et al.’s study supports the side-lying position especially for those who have given birth via cesarean section to achieve greater comfort and satisfaction and optimal infant feeding behaviors [34]. Thus, the mode of birth would seem to be an important factor to consider when adopting positions for breastfeeding.

In another study exploring the intensity of pain of primiparous women after giving birth via cesarean section, the intensity of pain after breastfeeding in the laid-back position group was lower than in the control group [35]. Nevertheless, this study was undertaken with a small sample size (13 women in each group), which may have influenced this difference in results. For instance, in an alternate study, the comfort of those following birth via cesarean section was found to be better in the breastfeeding side-lying position than the cradle position [36]. Yet, Palmer et al. (2019) highlighted that having previous breastfeeding experience can affect satisfaction with breastfeeding in subsequent experiences [37]. Conversely, studies have shown that the side-lying position during breastfeeding is suitable for those who are tired, or those who have had a cesarean section, or had experienced difficult birth, or when sitting is uncomfortable [38–40]. Thus, individual assessment will be important in all cases.

Having a favorable position and attachment causes effective transfer of milk and emptying of the breast and ensures pain-free feeding [20]. Both breastfeeding positions examined in the current study are modified positions that, according to studies, create the optimal technique and attachment bonding [20, 41, 42]. This evidence is also confirmed by the meta-analysis conducted by Dias and colleagues [43]. As both breastfeeding positions examined in the present study are related to creating a suitable attachment, it is assumed that they had the same effect on the occurrence of breast concerns in both groups.

Elsewhere, breast concerns in the laid-back position group have been found to be significantly less than in other positions [20]. Differences in results may be due to the difference in the measurement of breast concerns. In Wang et al.’s meta-analysis, the nipple trauma and its pain were measured to be less in the group using the laid-back breastfeeding position than in the group using traditional breastfeeding positions [24]. The low quality of the reviewed articles in the meta-analysis and the unreliability of its results may somewhat account for the differences noted between the present study and the meta-analysis [24].

With reference to milk insufficiency concerns, a systematic review of 27 articles by Huang et al. (2022) was conducted with the aim of determining the factors affecting the feeling of insufficient milk supply. The results demonstrated that there are many effective factors relating to milk insufficiency concerns such as a lack of education on EBF, absence of skin-to-skin contact during the first hours of birth, delay in starting the first breastfeed, being primiparous, and not having self-efficacy or sufficient knowledge about breastfeeding [44]. Education during pregnancy and after childbirth can reduce breastfeeding problems and increase the feeling of milk sufficiency [45]. In the present study, breastfeeding education and support was broadly the same for both groups and could explain the lack of statistically significant differences between the two groups in terms of milk insufficiency concerns.

In relation to neonatal concerns, there have similarly been no significant differences found in breast attachment scores between those breastfeeding in the laid-back and side-lying positions following birth via cesarean section elsewhere [23]. Similarly, no statistically significant difference was found in terms of breastfeeding outcomes (lactation status, attachment, sucking and swallowing) between those breastfeeding in the L-shaped and side-lying positions following birth via cesarean Sect. [33]. Yet, a meta-analysis demonstrated that those breastfeeding in the laid-back position had a higher frequency of attachment and positioning of the infant to the breast when compared to those breastfeeding in traditional positions [24]. Further research will be required with larger sample sizes to ascertain why such discrepancies may occur.

In several articles, education in relation to optimal breastfeeding techniques has been highlighted as an effective factor in enabling the infant to latch onto the breast effectively [41, 42]. In the present study, both breastfeeding positions facilitated infants to latch on to the breast effectively. As such, these positions could usefully be included in such education in future.

In relation to process concerns, studies have shown that professional and individual support and counseling are effective in increasing maternal self-confidence and improving the breastfeeding process [46–49]. Our results report no statistically significant difference in the mean score of breastfeeding experience in primiparous women at the time of discharge or at 1 or 2 weeks after childbirth. Negative breastfeeding experiences are an important risk factor for why breastfeeding may end early [50]. Breastfeeding problems can end breastfeeding earlier than planned [51], and previous breastfeeding experience can influence the breastfeeding of future children [52–54]. Previous positive breastfeeding experiences in particular can increase self-confidence and self-efficacy for breastfeeding subsequent children [55]. As such, it will be important to optimize all breastfeeding experiences in pursuit of improved maternal and child health globally.

### Limitations and suggestions for future research

Misconceptions about breastfeeding and individual differences in reporting were mitigated somewhat by randomization and sample selection. Future research may adopt observational tools to measure the problems experienced in breastfeeding more effectively. Another limitation of the study may be non-compliance with the intervention provided to participants in both groups and the use of other breastfeeding positions. Education and support should be adopted more widely and evaluated more robustly in pursuit of improved outcomes and experiences both during and after birth. Another limitation of this study is that it was conducted in only one center. Future studies should be multi-center in their design.

## Conclusion and implications for practice

No statistically significant differences were identified in terms of the mean score of maternal concerns, breast concerns, milk insufficiency concerns, neonatal concerns, process concerns, and breastfeeding experience between those who breastfed in the laid-back position, and those who breastfed in the cradle position at either 1 or 2 weeks following childbirth. Thus, these two breastfeeding positions evidentially have the same effect on the breastfeeding experience of primiparous (cisgender) women. Consequently, it is better to offer choice in breastfeeding positions and individualized approaches. Fundamentally, general breastfeeding education and counseling are more effective in optimizing success in breastfeeding than education on any one breastfeeding position in particular.

## Supplementary Information

**Additional file 1.**

**Additional file 2: Supplementary figure 1.** Comparison the mean of maternal concerns scores between the two groups at the times of hospital discharge, and one and two weeks after childbirth.

**Additional file 3: Supplementary figure 2.** Comparison the mean of breast concern scores between the two groups at the times of hospital discharge, and one and two weeks after childbirth.

**Additional file 4: Supplementary figure 3.** Comparison the mean of milk insufficiency concern scores between the two groups at the times of hospital discharge, and one and two weeks after childbirth.

**Additional file 5: Supplementary figure 4.** Comparison the mean of process concern scores between the two groups at the times of hospital discharge, and one and two weeks after childbirth.

**Additional file 6: Supplementary figure 5.** Comparison the mean of neonatal concerns scores between the two groups at the times of hospital discharge, and one and two weeks after childbirth.

## Data Availability

No additional data available due to confidentiality restrictions.
