# Supplementary material for: Comparing the effects of breastfeeding in the laid-back and cradle position upon the experiences of primiparous women: a parallel randomized clinical trial
Source: Trials. 2023 Feb 13;24:109. doi: 10.1186/s13063-023-07143-0 (PMC9926697; doi:10.1186/s13063-023-07143-0)
Supplement: Supplementary file 4 — Additional file 4: Supplementary figure 3. Comparison the mean of milk insufficiency concern scores between the two groups at the times of hospital discharge, and one and two weeks after childbirth. [file 13063_2023_7143_MOESM4_ESM.docx]

**Suplementry figure 3:** Comparison the mean of milk insufficiency concern scores between the two groups at the times of hospital discharge, and one and two weeks after childbirth
